# Supplementary material for: Association between the risk of malnutrition and functional capacity in patients with peripheral arterial disease: A cross-sectional study
Source: PLoS One. 2022 Sep 9;17(9):e0273051. doi: 10.1371/journal.pone.0273051 (PMC9462727; doi:10.1371/journal.pone.0273051)
Supplement: Attachment — Submitted filename: Yameng June 23.Comments to the Authors.docx [file pone.0273051.s003.docx]

Comments to the Authors

The authors are commended for their work.

For the comments 4-6, I suggest the authors make some supplements in the introduction section.
