# Supplementary material for: Association between the risk of malnutrition and functional capacity in patients with peripheral arterial disease: A cross-sectional study
Source: PLoS One. 2022 Sep 9;17(9):e0273051. doi: 10.1371/journal.pone.0273051 (PMC9462727; doi:10.1371/journal.pone.0273051)
Supplement: Attachment — Submitted filename: Response to Reviewers.docx [file pone.0273051.s004.docx]

July 2022

Dear Editor

The revised version of the manuscript entitled "Association between the risk of malnutrition and functional capacity in patients with peripheral arterial disease: A cross-sectional study" is attached.

We have revised the manuscript following the reviewers' comments and have included changes to the manuscript highlighted in blue text in the marked version of the manuscript. We hope you find this article suitable for publication. Thank you for your time and consideration.

Reviewer #1: 

2. Abstract line 45: Typo, change “analyzes” to “analyses.”

Thank you very much for the observation. It has been changed in the text. (Line 43).

3. Introduction: PAD is understood to be referring to atherosclerotic plaque development in the lower limbs. Thus consider changing the opening sentence to “…which results in partial or total obstruction in the arteries of the lower limbs.” and delete “…especially in the lower extremities.”

We have changed it according to the reviewer's suggestion. (Line 57).

4. Please include a hypothesis at the end of the Introduction section.

As suggested, we included the study hypothesis at the end of the introduction. (Line 81).

5. Page 5, line 10: “were” is repeated please delete.

Yes, we deleted it. (Line 105 - 107).

6. Page 5 and 6, lines 130-131: Delete the level of accuracy of height and weight measurements. It is awkward as written so if you do want to keep it please revise to clarify.

We followed the reviewer's important suggestion and removed it from the text.

7. Page 6 lines 141-142: Bad grammar in the sentence starting with “The data are…” Please revise.

Thank you, we modified the text. (Line 133 – 136).

8. Page 6 line 151: Why is there a bracket at the end of the sentence ending in “…stand test.”?

Yes, we changed. (Line 143).

9. Page 7 line157: I thought both gender were included so please change the term “his” to include both. Also in the same sentence the statement “…and the time was timed.” is awkward. Did the authors mean and the total time was recorded?

Thank you for this consideration. We have revised the grammar of this paragraph. (Line 147 – 149).

10. “His” seems to continue to be used which needs to be changed.

Yes, we changed.

11. Line 219: missing a period.

Thank you, we changed. (Line 211).

12. It would be helpful for the raw/continuous values of the outcomes were reported (e.g., 6MWT, SPPB, WIQ scores, etc).

Thanks for the comment. The 6MWT is presented in raw and relative data. However, the WIQ and SPPB tests are scored based on calculations.

Reviewer #2: Comments to the Authors

The authors are commended for their work.
For comments 4-6, I suggest the authors make some supplements in the introduction section.

We appreciated the reviewer's suggestions and made the necessary changes.
