# Supplementary material for: Association between the risk of malnutrition and functional capacity in patients with peripheral arterial disease: A cross-sectional study
Source: PLoS One. 2022 Sep 9;17(9):e0273051. doi: 10.1371/journal.pone.0273051 (PMC9462727; doi:10.1371/journal.pone.0273051)
Supplement: Attachment — Submitted filename: Yameng. August.1.Comments to the Authors.docx [file pone.0273051.s005.docx]

Comments to the Authors

The authors are commended for their work.

1. line 48: please use the same term. Change “ usual 4-meter walk test” to “gait speed test”.
2. Line 79: please add more details to increase the scientific evidence to emphasize the importance of this study.
3. For the practical implications, you authors could not concluded that being at risk of malnutrition can lead to a significant decline in walking capacity and lower limb strength. Please change this description.
